# Axonal transport defects are a common phenotype in *Drosophila* models of ALS

**DOI:** 10.1093/hmg/ddw105

**Published:** 2016-04-07

**Authors:** Katie R. Baldwin, Vinay K. Godena, Victoria L. Hewitt, Alexander J. Whitworth

**Affiliations:** 1 Department of Biomedical Sciences, University of Sheffield, Sheffield S10 2TN, UK; 2 Medical Research Council Mitochondrial Biology Unit, Cambridge Biomedical Campus, Hills Road, Cambridge CB2 0XY, UK

## Abstract

Amyotrophic lateral sclerosis (ALS) is characterized by the degeneration of motor neurons resulting in a catastrophic loss of motor function. Current therapies are severely limited owing to a poor mechanistic understanding of the pathobiology. Mutations in a large number of genes have now been linked to ALS, including *SOD1*, *TARDBP* (*TDP-43*)*, FUS* and *C9orf72.* Functional analyses of these genes and their pathogenic mutations have provided great insights into the underlying disease mechanisms. Defective axonal transport is hypothesized to be a key factor in the selective vulnerability of motor nerves due to their extraordinary length and evidence that ALS occurs as a distal axonopathy. Axonal transport is seen as an early pathogenic event that precedes cell loss and clinical symptoms and so represents an upstream mechanism for therapeutic targeting. Studies have begun to describe the impact of a few pathogenic mutations on axonal transport but a broad survey across a range of models and cargos is warranted. Here, we assessed the axonal transport of different cargos in multiple *Drosophila* models of ALS. We found that axonal transport defects are common across all models tested, although they often showed a differential effect between mitochondria and vesicle cargos. Motor deficits were also common across the models and generally worsened with age, though surprisingly there was not a clear correlation between the severity of axonal transport defects and motor ability. These results further support defects in axonal transport as a common factor in models of ALS that may contribute to the pathogenic process.

## Introduction

Amyotrophic lateral sclerosis (ALS) is a typically adult onset progressive neurodegenerative disorder and the most common form of motor neuron disease. It is characterized by the loss of both the upper and lower motor neurons representing a catastrophic loss of motor function. The condition is fatal, usually due to respiratory failure, with an average life expectancy of 3–5 years from diagnosis (1). There is no cure and current therapies are severely limited owing to a poor mechanistic understanding of the pathobiology. Although the majority of ALS cases is sporadic, ∼10% are monogenic, familial forms. A large number of genes have now been linked to ALS, including *SOD1*, *TARDBP* (*TDP-43*)*, FUS* and *C9orf72.* Functional analyses of these genes and pathogenic mutations have provided great insight into the underlying disease mechanisms (1,2).

TDP-43 is a multi-functional DNA/RNA-binding protein that shuttles between the nucleus and cytoplasm (3). In the nucleus, it plays many roles in transcription and RNA processing (4–7). In the cytoplasm, TDP-43 localizes to stress granules, P-bodies and RNA transport granules, and is involved in the regulation and spatial distribution of RNAs (8–12). FUS is also a DNA/RNA-binding protein that undergoes nuclear/cytoplasmic shuttling, with functions in RNA processing (13,14). The targets of TDP-43 and FUS RNA processing number in the thousands in animal models, although there appears to be only limited overlap (13,15). However, TDP-43 and FUS targets show some functional commonality suggesting defects in TDP-43 or FUS function could lead to common pathogenic outcomes (15,16).

Expansion of a hexanucleotide repeat GGGGCC in the first intron of *C9orf72* is the most common genetic cause of ALS (17,18). Bidirectional transcription of these repeats forms nuclear RNA foci which sequester RNA-binding proteins (RBPs) (17,19–24). Moreover, these repeats undergo repeat associated non-ATG (RAN) translation, giving rise to a series of dipeptide repeat proteins (DPRs). These DPRs have a high propensity to aggregate and form inclusions in *C9orf72* associated patient tissue (25–28). Recent research has suggested that pathogenicity is specifically associated with arginine containing DPRs (29,30).

These insights into the molecular causes of ALS, however, still do not provide a clear rationale for the selective cell-type vulnerability implicit in the disease. A defining feature of motor neurons is the extraordinary length of their axons. This characteristic, coupled with evidence indicating that ALS occurs as a distal axonopathy, has led to defective axonal transport being implicated as a key initiating contributor to the selective vulnerability of motor nerves. Emerging evidence has begun to highlight the impact of pathogenic mutations on axonal transport. For example, expression of mutant SOD1 in motor nerves causes axonal transport defects as an early pathology that precedes cell loss and clinical symptoms (31,32). Multiple types of cargo are affected including mitochondria (33), neurofilaments (32) and vesicles (34). Overexpression of the wild-type and several pathogenic mutant TDP-43 proteins leads to early onset mitochondrial transport dysfunction (35,36); however, this was not observed in another study (8). Thus, further work remains to be done to elucidate the relationship between axonal transport and degeneration in TDP-43 and other ALS models.

Here, we analyzed fast axonal transport in larval motor neurons of *Drosophila* models of *TARDBP* (*TDP-43*), *FUS* and *C9orf72*. We also analyzed the effect of loss-of-function mutants of the *Drosophila* orthologs of *TDP-43* and *FUS*, *TBPH* and *caz*, respectively. The motor activities of larvae and adults in these models were assessed to correlate potential defects in axonal transport with locomotor deficits. We found that axonal transport defects are common across all models tested, although they often showed a differential effect between mitochondria and vesicle cargos. Motor deficits were also common across the models, though did not always show a clear correlation between the severity of axonal transport defects and motor ability.

## Results

### Gain and loss of *TDP-43* function have differential effects on axonal transport

**Figure 1.**
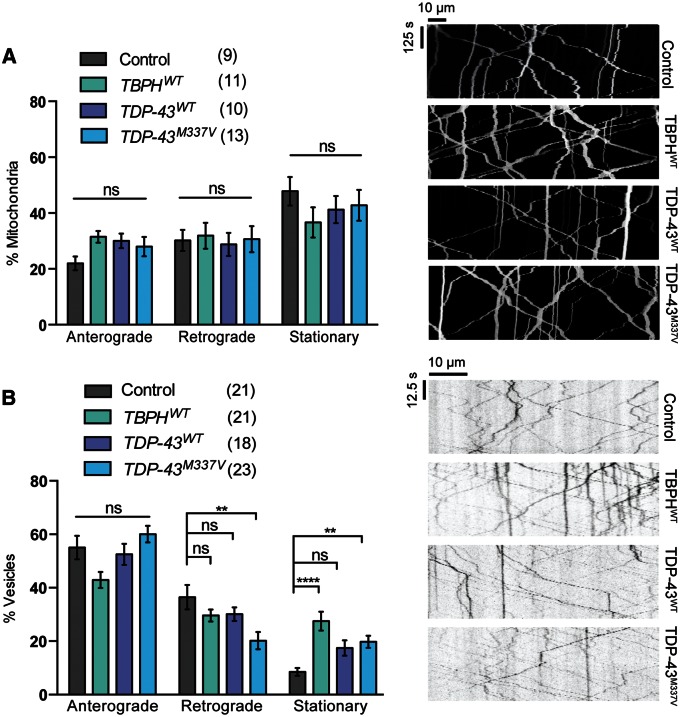
Overexpression of *TDP-43* affects the axonal transport of vesicles but not mitochondria. Live imaging analysis of (**A**) mitochondrial transport or (**B**) NPY containing vesicles in *Drosophila* motor axons overexpressing either *Drosophila TBPH* or human *TDP-43* variants. Mitochondria are marked by *UAS-mito.GFP* and vesicles by *UAS-NPY.GFP*, driven by *CCAP-GAL4*. The number in brackets indicates the number of movies analyzed. Representative kymographs of the indicated genotypes are shown. Control genotype; (A) *CCAP-GAL4/+*; *UAS-mito.GFP/UAS-lacZ*, (B) *CCAP-GAL4/+*; *UAS-NPY.GFP/UAS-lacZ*. Statistical analysis was performed using one-way ANOVA with Sidak’s multiple comparison test: **P < 0.01, ****P < 0.0001. Charts show mean ± SEM.

Mutations in *TDP-43* cause ALS in an autosomal dominant manner and likely impact on neuronal function in a multitude of ways. We first analyzed whether ectopic expression of a pathogenic variant of *TDP-43*, M337V, caused any disruption of axonal transport in *Drosophila* motor nerves in comparison to *TDP-43^WT^*. To minimize the likelihood of artifacts from different expression levels or from disruption of genomic insertion, we made use of transgenes generated in the same integration site and expressing at equivalent levels (see Materials and Methods). Somewhat surprisingly no significant effect on mitochondrial transport was observed for any of the transgenes (Fig. 1A). However, when we analyzed a different transport cargo—vesicles loaded with GFP-fused neuropeptide Y (NPY.GFP)—although *TDP-43^WT^* did not cause any significant defects, *TDP-43^M337V^* expression caused a decrease in motility, with a concomitant significant increase in the stationary fraction (Fig. 1B). Overexpression of the fly homolog of *TDP-43*, named *TBPH*, also interfered with the transport of vesicles but not mitochondria (Fig. 1).

**Figure 2.**
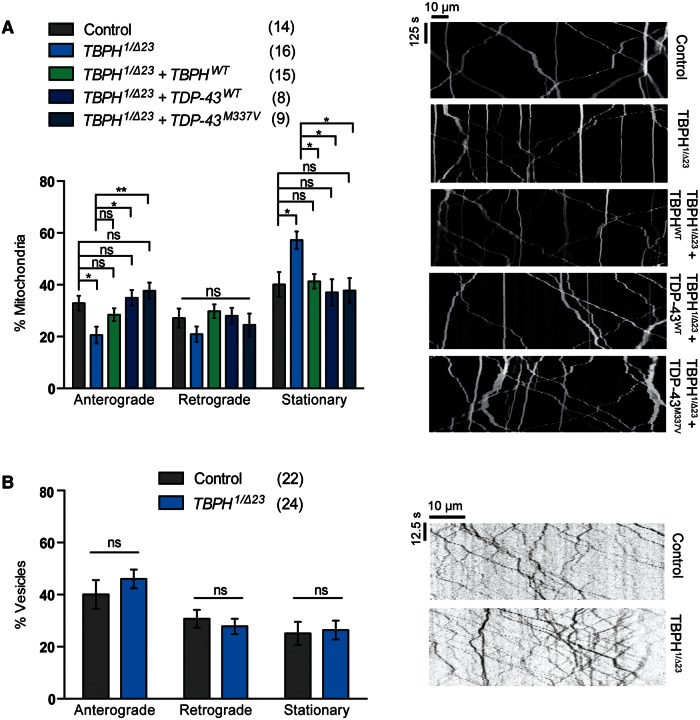
Loss of *TBPH* inhibits axonal transport of mitochondria but not vesicles. Live imaging analysis of (**A**) mitochondrial transport or (**B**) NPY containing vesicles in *Drosophila* motor axons. Transheterozygous *TBPH* mutants were analyzed alone or in combination with transgenic expression of wild-type *TBPH*, or wild-type or a pathogenic variant of *TDP-43*. The number in brackets indicates the number of movies analyzed. Representative kymographs of the indicated genotypes are shown. Control genotype; (A) *CCAP-GAL4/+; UAS-mito.GFP/UAS-lacZ*, (B) *CCAP-GAL4/+; UAS-NPY.GFP/+*. Statistical analysis was performed using one-way ANOVA with Sidak’s multiple comparison test: *P < 0.05, **P < 0.01. Charts show mean ± SEM.

We next addressed whether *TDP-43* normally plays a role in axonal transport by examining whether loss of the endogenous *TBPH* disrupted transport. We used a trans-heterozygous combination of the independently derived *TBPH^1^* and the *TBPH^Δ23^* deletion mutants to avoid potential genetic background effects. Loss of *TBPH* caused an overall increase in the stationary fraction of mitochondria (Fig. 2A). Although there was an observable decrease in both anterograde and retrograde transport, this only reached significance in the anterograde direction. In contrast, we found no impact of loss of *TBPH* on vesicle transport (Fig. 2B).

Importantly, the disruption of mitochondrial transport could be completely rescued by re-expression of a *TBPH^WT^* transgene (Fig. 2A), verifying that this effect is a direct consequence of the genetic loss of *TBPH*. Interestingly, the loss of mitochondrial transport was also rescued by the ectopic expression of human *TDP-43^WT^* and by the ALS-linked variant *TDP-43^M337V^*. This cross-species rescue is consistent with previous reports and reflects their close homology, but also reveals that the pathogenic variant can broadly function as normal. Overall, these results indicate that dysregulated *TDP-43* expression can affect axonal transport but cargoes are affected differently, and suggest that the pathogenic mutation may increase the susceptibility to disease.

### Axonal transport disruption in *TDP-43* models correlates with behavioral deficits

**Figure 3.**
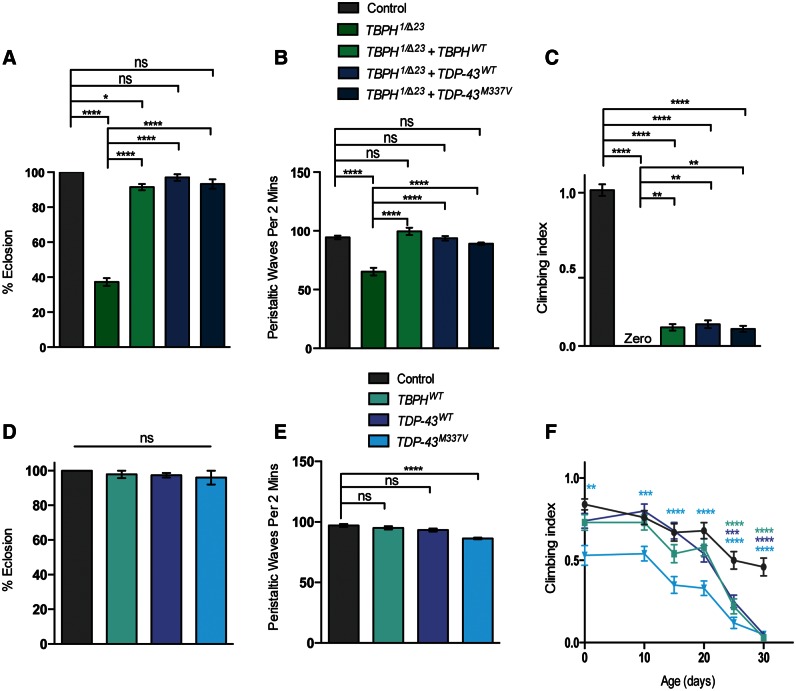
Gain and loss of *TBPH/TDP-43* cause motor behavioral defects. *TBPH* mutants were analyzed alone or in combination with transgenic expression of wild-type *TBPH*, or wild-type or a pathogenic variant of *TDP-43*, were assayed for (**A**) eclosion, (**B**) larval crawling, or (**C**) adult climbing ability. Transgenes were expressed in motor neurons via the *D42-GAL4* driver. Animals overexpressing *TBPH* or *TDP-43* variants in a wild-type background were assayed for (**D**) eclosion, (**E**) larval crawling, or (**F**) adult climbing ability. *N* (animals) ≥ 100 (A, D), ≥20 (B, E), ≥50 (C, F). Control genotypes are *TBPH^1^*/+; *D42-GAL4*/+ (A–C) and *D42-GAL4/UAS-lacZ* (D–F). The mutant genotype carries heterozygous *D42-GAL4* in the background. Data in (C) and (F) are normalized to control. Statistical analysis was performed using one-way ANOVA with Sidak’s multiple comparison test, except (C) and (F) which were analyzed by Kruskal–Wallis non-parametric test with Dunn’s correction: *P < 0.05, **P < 0.01, ***P < 0.001, ****P < 0.0001. Comparisons in (C) are with control. Charts show mean ± SEM.

A prime motivation for this study was to correlate disruptions in axonal transport to decline in motor neuron function; the cardinal clinical feature of ALS. Thus, flies were examined for their neuromuscular function using a number of assays. One indicator of severe motor dysfunction is the inability of mutant flies to eclose from the pupal case after undergoing metamorphosis. For *TBPH* mutants, <40% of pupae produce viable adults (Fig. 3A), all of whom died within 5 days (37,38). As a more direct measure, we analyzed larval crawling and found that *TBPH* mutants exhibit a significant motor deficit (Fig. 3B). Furthermore, the few adult mutants that do eclose are uncoordinated and fail to register any climbing capacity (Fig. 3C). These phenotypes could all be rescued by the re-expression of either fly or human *TDP-43* variants. For eclosion and larval crawling, the phenotypes were rescued back to control levels; however, the adult locomotion showed very limited rescue suggesting that cell types other than motor neurons substantially contribute to this severe phenotype.

In contrast, the overexpression of the *TBPH*/*TDP-43* has relatively modest effects on motor function. Overexpression of wild-type *TBPH*/*TDP-43* does not perturb viability, larval crawling or climbing ability in young adult flies (Fig. 3D–F). However, the ectopic expression of *TDP-43^M337V^* caused a modest loss of larval locomotion but had a more pronounced effect on adult climbing. Interestingly, when analyzed with age, the climbing behavior in all three conditions progressively worsened, with the effect of wild-type *TBPH*/*TDP-43* overexpression eventually mirroring that of the pathogenic mutant (Fig. 3F). Overall, these findings support a relationship between the observed transport defects and aberrant motor behavior.

### Loss or gain of *caz* causes broad axonal transport disruption but gain of *FUS* specifically disrupts transport of vesicles

**Figure 4.**
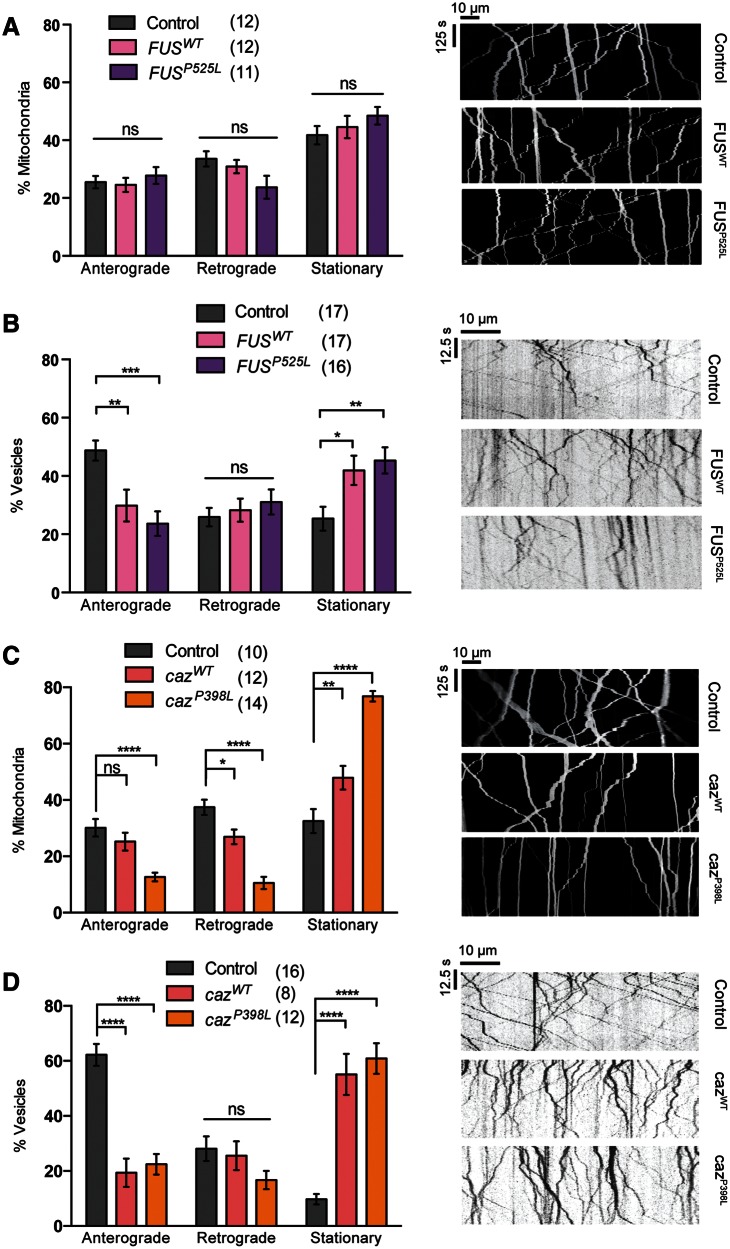
Overexpression of *FUS* or *caz* differentially inhibits mitochondrial and vesicle transport. Live imaging analysis of (**A**, **C**) mitochondrial transport and (**B**, **D**) vesicle transport in *Drosophila* motor axons. Animals overexpressing wild-type or a pathogenic variant of *FUS* (A and B) or the *Drosophila* homolog *caz* (C and D) were analyzed. Representative kymographs of the indicated genotypes are displayed. The number in brackets indicates number of movies analyzed. Control genotype; (A, B) *CCAP-GAL4/+*; *UAS-mito.GFP/UAS-lacZ*, (C, D) *CCAP-GAL4/+*; *UAS-NPY.GFP/UAS-lacZ.* Statistical analysis was performed using one-way ANOVA with Sidak’s multiple comparison test: *P < 0.05, **P < 0.01, ***P < 0.001, ****P < 0.0001. Charts show mean ± SEM.

We next sought to broaden our analysis and determine whether axonal transport deficits were apparent in other models of ALS; thus, we analyzed *Drosophila* models of *FUS* and its homolog *cabeza* (*caz*). Ectopic expression of *FUS^WT^*or a pathogenic variant *FUS^P525L^* caused no disruption to mitochondrial transport (Fig. 4A). However, the expression of both *FUS* variants led to an increase in the stationary fraction of vesicles (Fig. 4B). Interestingly, this appeared to be due to a selective decrease in anterograde movement. In comparison, overexpression of *caz^WT^* and the pathogenic equivalent *caz^P398L^* inhibited the transport of both mitochondria and vesicles (Fig. 4C and D). Notably, although both *caz* variants had a similar effect on vesicle transport, the pathogenic variant had a substantially more marked effect on mitochondrial transport.

**Figure 5.**
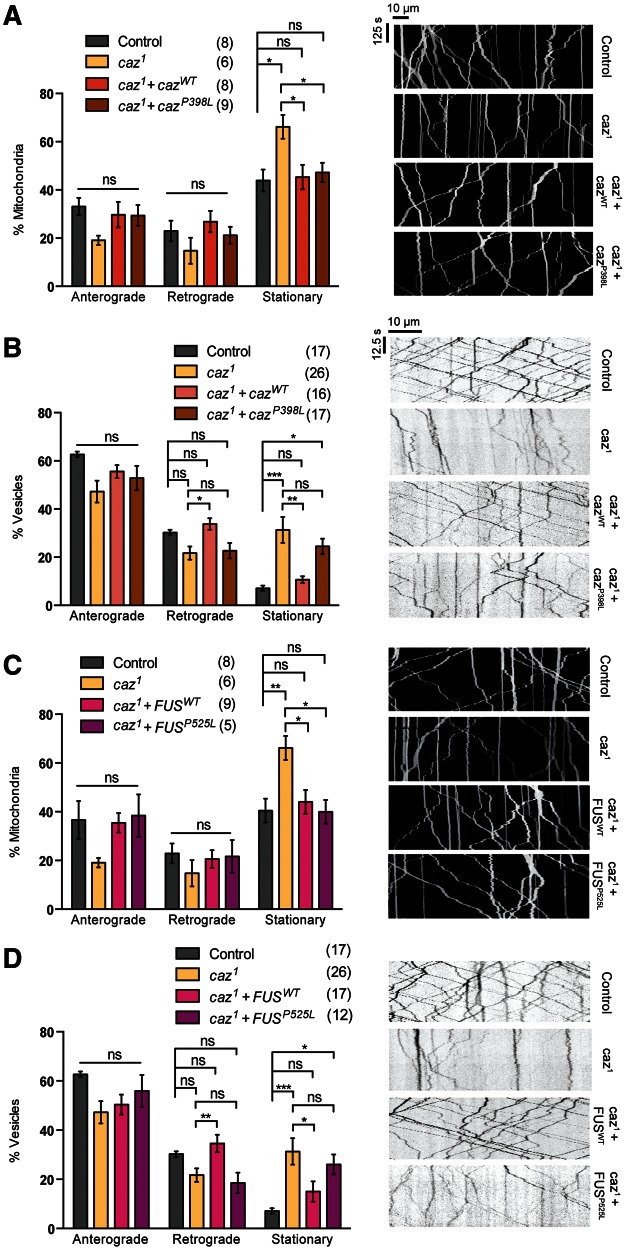
Loss of *caz* inhibits mitochondrial and vesicle transport. Live imaging analysis of (**A**, **C**) mitochondrial transport and (**B**, **D**) vesicle transport in *Drosophila* motor axons. *caz^1^* mutants were analyzed alone or in combination with transgenic expression of wild-type or a pathogenic variant of *caz* (A and B) or *FUS* (C and D) were analyzed. Representative kymographs of the indicated genotypes are shown. The number in brackets indicates the number of movies analyzed. Control genotype; (A, B) *CCAP-GAL4/+; UAS-mito.GFP/lacZ*, (C, D) *CCAP-GAL4/+; UAS-NPY.GFP/lacZ.* Statistical analysis was performed using one-way ANOVA with Sidak’s multiple comparison test: *P < 0.05, **P < 0.01, ***P < 0.001. Charts show mean ± SEM.

Analyzing the *caz^1^* null mutant, we found a significant decrease in the transport of both mitochondria and vesicles (Fig. 5A and B). These phenotypes could be completely rescued by the re-expression of *caz^WT^* (Fig. 5A and B). It is worth noting that although vesicle transport was rescued by *caz^WT^* under standard conditions (i.e. raised at 25 °C), animals needed to be raised at 29 °C, increasing transgene expression levels, to see a complete rescue of the mitochondrial transport. Interestingly, the re-expression of the pathogenic variant, *caz^P398L^*, had differential rescuing effects. Although it was able to rescue the mitochondrial transport defect, it was unable to rescue the disruption in vesicle transport. Similarly, expression of *FUS^WT^* was able to rescue the *caz^1^* mutant transport deficit of mitochondria and vesicles (Fig. 5C and D). We also saw a strikingly similar effect from the *FUS^P525L^* variant as observed for the equivalent *caz^P398L^*; the mitochondrial transport was rescued but vesicle transport was not (Fig. 5C and D).

### Axonal transport disruption in *FUS/caz* models correlates with behavioral deficits

**Figure 6.**
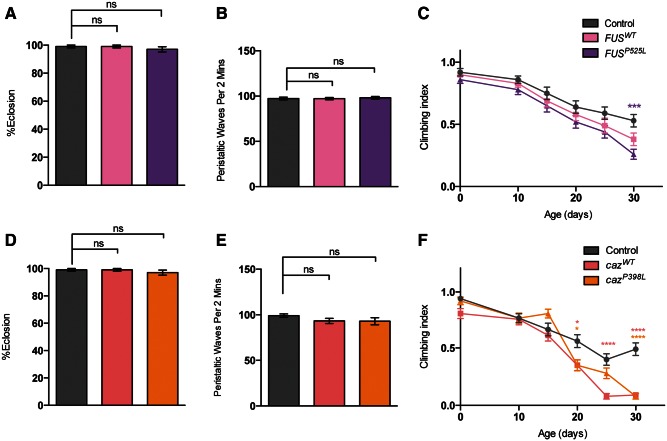
Expression of *FUS/caz* variants has limited effect on motor behavior. Animals overexpressing wild-type or a pathogenic variant of *FUS* (**A–C**) or *caz* (**D–F**) in a wild-type background were assayed for (A and D) eclosion, (B and E) larval crawling, or (C and F) adult climbing ability, driven by *D42-GAL4*. *N* (animals) ≥ 100 (A, D), ≥20 (B, E), ≥50 (C, F). Control genotype is *D42-GAL4/UAS-lacZ.* Data in (C) and (F) are normalized to control. Statistical analysis was performed using one-way ANOVA with Sidak’s multiple comparison test, except (C) and (F) which were analyzed by Kruskal–Wallis non-parametric test with Dunn’s correction: *P < 0.05, **P < 0.01, ***P < 0.001, ****P < 0.0001. Charts show mean ± SEM.

Analyzing the effects of *FUS/caz* mutations on motor ability, we found that overexpression of either *FUS* or *caz* variants had almost no effect on eclosion, larval crawling or young adult locomotion (Fig. 6A–F). However, climbing ability began to decline more rapidly in adults overexpressing the pathogenic *FUS^P525L^* (Fig. 6C). Overexpression of either form of *caz* significantly perturbed motor ability to a similar extent (Fig. 6F).

**Figure 7.**
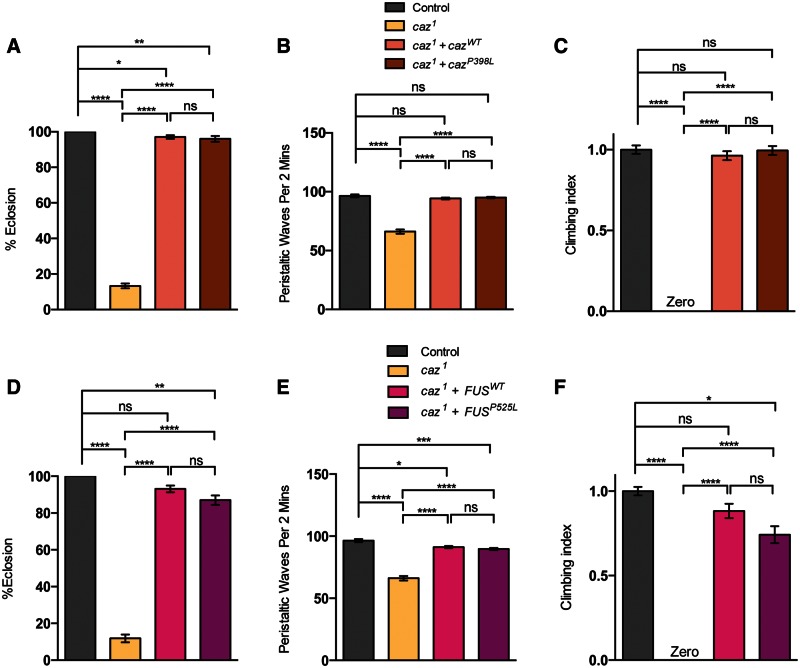
Loss of *caz* causes reduced motor ability. *caz^1^* mutants were analyzed alone or in combination with transgenic expression of wild-type or a pathogenic variant of *caz* or *FUS*, driven by *D42-GAL4*. The indicated genotypes were assayed for (**A** and **D**) eclosion, (**B** and **E**) larval crawling, or (**C** and **F**) adult climbing ability. *N* (animals) ≥ 100 (A, D), ≥20 (B, E), ≥50 (C, F). Control genotype is *D42-GAL4/UAS-lacZ.* The mutant genotype carries heterozygous *D42-GAL4* in the background. Data in (C) and (F) are normalized to control. Statistical analysis was performed using one-way ANOVA with Sidak’s multiple comparison test, except (C) and (F) which were analyzed by Kruskal–Wallis non-parametric test with Dunn’s correction: *P < 0.05, **P < 0.01, ***P < 0.001, ****P < 0.0001. Charts show mean ± SEM.

*caz^1^*mutants had a dramatic reduction in eclosion (Fig. 7A), as reported previously (39). Larval crawling was also significantly reduced, whereas the escaper adult mutants failed to register any climbing activity, reminiscent of the *TBPH* mutants (Fig. 7B and C). These phenotypes could be substantially rescued by the re-expression of either *caz^WT^* or *FUS^WT^* as previously reported (39). The pathogenic variant was also able to rescue these phenotypes to a level similar to the wild-type version (Fig. 7A–F).

### *caz* overexpression rescues *TBPH* motor function but not axonal transport

**Figure 8.**
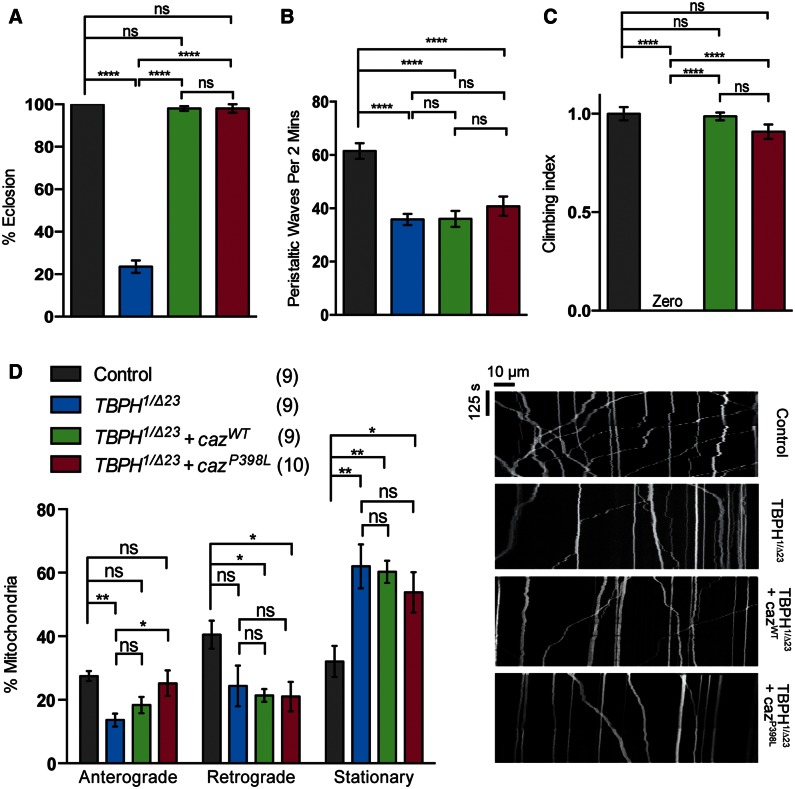
Overexpression of *caz* can rescue behavioral but not transport phenotypes in *TBPH* mutants. *TBPH* mutants were analyzed alone or in combination with transgenic expression of *caz* variants and assayed for (**A**) eclosion, (**B**) larval crawling and (**C**) adult climbing ability. (**D**) Live imaging analysis of mitochondrial transport in *Drosophila* motor axons. The number in brackets indicates the number of movies analyzed and representative kymographs of the indicated genotypes are shown. *N* (animals) ≥ 100 (A), ≥20 (B), ≥50 (C). Control genotypes; (A, B, C) *D42-GAL4/UAS-lacZ*, (D) *CCAP-GAL4/+; UAS-mito.GFP/+*. The mutant genotype carries heterozygous *D42-GAL4* in the background. Data in (C) are normalized to control. Statistical analysis was performed using one-way ANOVA with Sidak’s multiple comparison test, except (C) which was analyzed by Kruskal–Wallis non-parametric test with Dunn’s correction: *P < 0.05, **P < 0.01, ****P < 0.0001. Charts show mean ± SEM.

*TDP-43* and *FUS*, and their fly orthologs, have been shown to genetically interact. The reduced viability and lifespan of *TBPH* mutants were rescued by neuronal overexpression of *caz*, whereas the locomotor deficit was partially rescued; however, overexpression of *TBPH* did not alter the *caz^1^* phenotypes (39). In agreement with these findings, we also saw a complete rescue of eclosion and adult climbing ability (Fig. 8A and C).

In light of this, we assayed whether the mitochondrial axonal transport defect (Fig. 5) was also rescued. Surprisingly, the transport defect in *TBPH* mutants was not rescued by overexpression of either *caz^WT^* or *caz^P398L^* (Fig. 8D). However, consistent with this we found that larval crawling was not rescued by *caz* expression (Fig. 8B). Therefore, it appears that although *TBPH* and *caz* act within a genetic pathway influencing viability and adult motor behaviors, this is not shared in the mechanisms that disrupt axonal transport.

### *C9orf72*-linked hexanucleotide repeats severely impair transport but only mildly disrupts behavior

**Figure 9.**
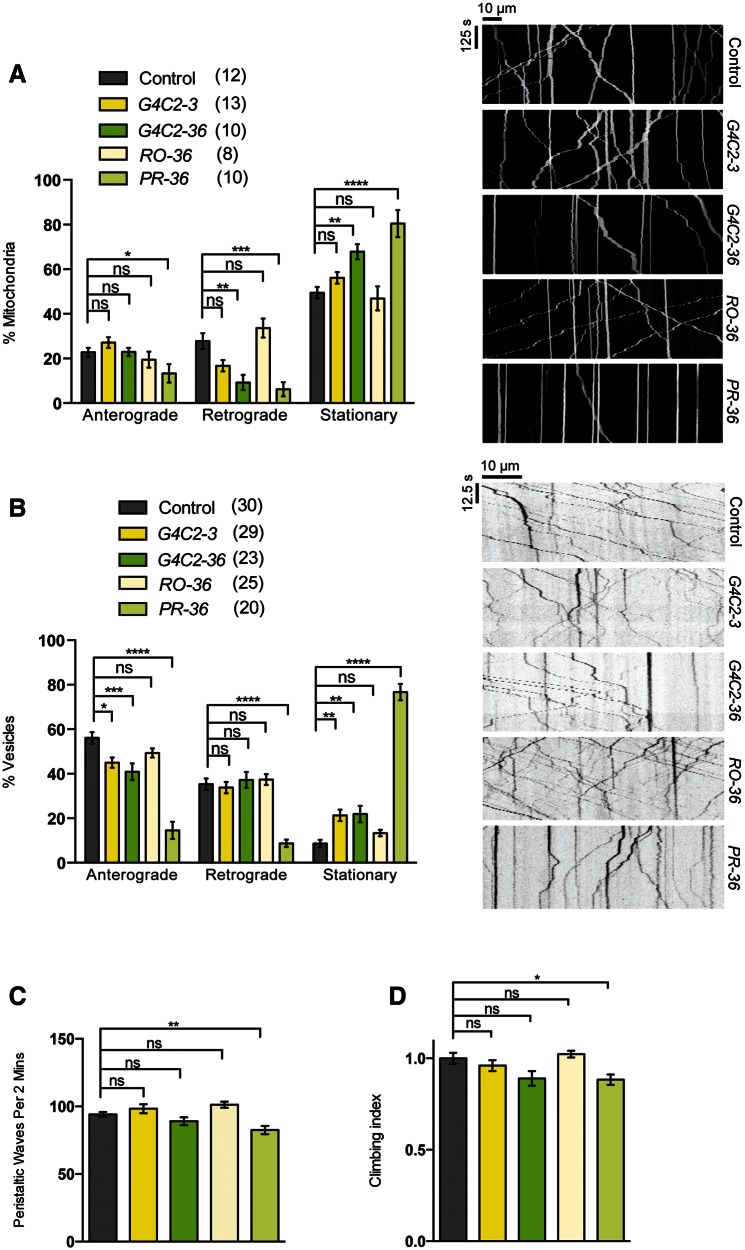
*C9orf72-*related dipeptide repeats inhibit axonal transport and cause a mild motor deficit. Animals expressing control length (*G4C2-3*) or expanded (*G4C2-36*) hexanucleotide repeats, expanded RNA-only (*RO-36*) or proline-arginine dipeptide repeats (*PR-36*) were analyzed for axonal transport of (**A**) mitochondria, (**B**) vesicles, or motor function as (**C**) larval crawling or (**D**) adult climbing ability. The number in brackets indicates the number of movies analyzed and representative kymographs of the indicated genotypes are shown. *N* (animals) ≥ 20 (C), ≥50 (D). Control genotypes; (A) *CCAP-GAL4/+;UAS-mito.GFP/+*, (B) *CCAP-GAL4/+; UAS-NPY.GFP/UAS-lacZ*, (C, D) *D42-GAL4/UAS-lacZ.* Data in (D) are normalized to control. Statistical analysis was performed using one-way ANOVA with Sidak’s multiple comparison test, except (D) which was analyzed by Kruskal–Wallis non-parametric test with Dunn’s correction: *P< 0.05, **P< 0.01, ***P < 0.001, ****P < 0.0001. Charts show mean ± SEM.

The third genetic model of ALS assessed in this study was the *C9orf72*-associated hexanucleotide (GGGGCC [G4C2]) repeats. Transgenic expression of a non-pathogenic repeat length (G4C2-3) had no effect on mitochondrial transport; however, expression of 36 repeats (G4C2-36), previously shown to cause neurotoxicity (30), caused a significant increase in the stationary fraction of mitochondria (Fig. 9A). To assess whether this was due to RNA toxicity or the production of arginine-containing dipeptide repeats, we analyzed animals expressing ‘RNA-only’ (RO-36) or proline–arginine encoding (PR-36) transcripts (30) and found that this effect was specifically attributable to the dipeptide repeats (Fig. 9A). Interestingly, vesicle transport was already slightly disrupted by G4C2-3, which was not substantially increased by G4C2-36 expression (Fig. 9B). Although the RO-36 repeats were again non-toxic, the expression of PR-36 caused a severe disruption of vesicle transport (Fig. 9B).

Despite the axonal transport phenotypes, neuromuscular function appears to be only mildly affected. There were no obvious effects on viability (data not shown), and the locomotor capacity of larvae and young adults was modestly affected only by PR-36 (Fig. 9C and D). Therefore, it seems that these disruptions to axonal transport are reasonably well tolerated in young flies, although a progressive aged-related decline in neuromuscular function was not tested.

## Discussion

In this study we sought to determine whether disruption of axonal transport is a common feature of ALS pathogenesis, which may underlie the disruption of neuromuscular function and loss of motor ability. The neuromuscular junction is highly energetically demanding and accordingly is dependent on correct distribution and function of mitochondria (40). Thus, disruption in mitochondrial transport has long been postulated to contribute to neuronal dysfunction, impacting on energy supply and calcium buffering capacity at the neuromuscular junction. Nonetheless, many other cellular components need to be transported to synapses to support proper functioning. Here, using previously characterized *Drosophila* models of ALS, we examined the transport of two different cargos and correlated any transport deficiencies with motor ability. Overall, we found that axonal transport deficits are a common feature in all the models analyzed here, but the severity and cargo selectivity differ substantially between models. Moreover, the severity of motor deficits does not always mirror the severity of axonal transport defects.

Gain and loss of *TDP-43*/*TBPH* exert differential effects on axonal transport. The overexpression of *TDP-43* variants did not affect mitochondrial axonal transport but impaired vesicle transport, whereas loss of *TBPH* caused a decrease in the transport of mitochondria but vesicle transport was unaffected. The relatively modest deficit in vesicle transport in the overexpression models correlated with modest locomotor deficits, whereas the loss of mitochondrial transport in *TBPH* mutants correlated with severe motor impairment.

Both gain and loss of function of *FUS*/*caz* affected axonal transport, though the effects were generally more widespread. Although the overexpression of *caz* variants affected both mitochondria and vesicle transport, overexpression of *FUS* variants only perturbed vesicle transport. Loss of *caz* substantially affected both vesicle and mitochondrial transport. The effects on motor ability were similar to *TDP-43*/*TBPH*; although the *caz* mutants exhibit severe developmental and locomotor defects, the overexpression models have only modest effects on adult locomotion that become more pronounced with age.

Axonal transport was also disrupted in the *C9orf72* models. The expanded pure G4C2-36 repeats caused a disruption of mitochondrial transport; however, the effect on the transport of vesicles is more complicated as the G4C2-3 repeats also slightly inhibit vesicles transport. Nevertheless, results from the RO-36 and PR-36 transgenes further support the view that toxicity of these expanded repeats is caused by DPR production and not ‘RNA toxicity’ as the RO-36 transgene appeared inert in all assays whereas the PR-36 transgene was highly toxic, substantially inhibiting mitochondrial and vesicle transport. Interestingly, expression of this transgene caused only modest motor deficits.

Thus, our data indicate that axonal transport deficits are a common feature in all the models analyzed here. Disruption of axonal transport has also been noted in several vertebrate models of ALS, most notably in models of SOD1 mutations and TDP-43 models. We chose to not investigate the impact of SOD1 mutations as they have been fairly well characterized in vertebrates, and have been found to affect neurofilaments (31,32), mitochondria (33) and vesicles (34). TDP-43 has been reported to modestly affect axonal transport of mitochondria (35), although, consistent with our observations, disruption of mitochondrial transport was not substantiated in another study (8). To our knowledge, our results are the first reporting an impact of FUS or G4C2-repeats on axonal transport. Hence, our findings confirm and extend the body of evidence indicating a general impact of axonal transport deficits in diverse models of ALS. It is also notable that in the vertebrate studies no clear pattern of disruption has emerged, further supporting a more generalized disruption of axonal transport.

Although this study has highlighted axonal transport deficits as a common feature in *Drosophila* models of ALS, axonal transport deficits are by no means specific to ALS because they have also been observed in other disease models. Axonal transport has been shown to be disrupted in models of Alzheimer’s disease (AD), polyglutamine (polyQ) diseases such as Huntington’s disease, peripheral neuropathies and Parkinson’s disease (PD). It is notable that the proteins in question in these disease models are all functionally quite different from each other and from the ALS-related disease factors studied here, indicating that diverse mechanisms can disrupt axonal transport. The models of AD implicate Tau-linked disruption of microtubules or dysregulation of kinases and phosphatases to impair transport (41). Huntingtin has been shown to interact with motor proteins via HAP-1; however, axonal transport disrupted is also disrupted by expanded polyQ in other disease contexts and thought to arise indirectly from blockages caused by aggregated polyQ (42). A wide array of mechanisms have been proposed for peripheral neuropathies but many center on cytoskeleton, molecular motors and disruption of mitochondrial distribution and function (43). Related to PD, an elegant mechanism has been elucidated by which PINK1 and Parkin specifically regulate the transport of mitochondria by regulating the turnover of the Miro–Milton complex that connects mitochondria to kinesin (44). More recently, mutations in LRRK2 have been shown interfere with axonal transport by influencing the acetylation state of microtubules, although the exact mechanisms are not clear (45). Thus, it is clear that disturbances to multiple and diverse cellular processes have the potential to disrupt axonal transport.

This study did not seek to determine the mechanism(s) of axonal transport defects but emerging evidence strongly implicates RNA dysregulation in TDP-43, FUS and G4C2-repeats mechanisms. TDP-43 and FUS function as part of ribonucleoprotein complexes in the regulation of RNA metabolism, and dysregulation leads to widespread effects on transcription and RNA splicing and transport. A growing consensus proposes that these RBPs sequester mRNAs in stress granules (46). Recent work has revealed that TDP-43 and related RBPs form stress granules via liquid–liquid phase separation (47). Moreover, pathogenic mutations enhance the propensity for RBPs to fibrilize within these granules, which is proposed to interfere with stress granule disassembly.

Both loss and gain of *TDP-43* and *FUS* result in neurodegenerative phenotypes (48–51), indicating that correct levels of these proteins are critical. Our results support the concept that the stoichiometry of ribonucleoprotein complexes is a key factor, and if the complex composition is altered functioning is impaired. Moreover, changes to the stoichiometry of ribonucleoprotein complexes may result in differential effects on their various targets; this may reflect the differences observed in the cargos that are affected by different mutations.

Both TDP-43 and FUS have been shown to bind and regulate RNAs of several motor proteins including KIF13 and KLC1 (7), and KIF5C, KIF1B and KIF3A (13,52), respectively. KIF13 is a kinesin 3 motor involved in vesicle transport, whereas KIF5C, KIF1B and KIF3A are involved in the axonal transport of mitochondria and vesicles. KLC1 is a kinesin light chain, which interacts with several kinesin heavy chain proteins. Interestingly, TDP-43 has also been reported to bind the mRNA of TRAK1 (13), homologous to *Drosophila* Milton, an adaptor protein that along with Miro specifically mediates the binding of mitochondria to the motor Kinesin 1 (53). Dysregulation of *TRAK1*/*milton* may underlie the mitochondria-specific effect observed in *TBPH* mutants.

Although it is early days for understanding the mechanism(s) by which *C9orf72* G4C2 repeat expansions cause disease, no clear picture is emerging that explains the selective neuronal vulnerability. The formation of RNA foci from the expanded G4C2 repeats again presents the possibility for wide-spread RNA dysregulation as the pathogenic cause, but this seems unlikely in light of the compelling observations from *Drosophila* that arginine-containing DPRs are highly neurotoxic whereas ‘RNA only’ repeats are not (30). Furthermore, recent reports have shown that inhibiting nuclear export of these repeats prevents much of the toxicity (54–56). Thus, the effects of cytoplasmic DPR toxicity may more indirectly affect sensitive cellular processes such as axonal transport. In summary, our findings support axonal transport defects as a shared phenomenon in multiple models of ALS, highlighting this as a potential common upstream target for therapeutic manipulation.

## Materials and Methods

### *Drosophila* genetics

*Drosophila* were raised under standard conditions at 25 °C on food consisting of agar, cornmeal, molasses and yeast unless otherwise stated. The UAS transgenes for *TBPH*, *TDP-43*, *caz* and *FUS*, which are all inserted in the attP2 landing site, as well as the *caz^1^* mutant were a gift from Brian McCabe (Columbia University) (51). The *TBPH^1^* line was a gift from Aaron Voigt (RWTH Aachen University) (57) and the *TBPH^Δ23^* a gift from Fabian Feiguin (ICGEB, Trieste) (38). All *C9orf72*-related transgenic lines, which are all inserted in the attP40 landing site, were a gift from Adrian Isaacs and Linda Partridge (University College London) (30). *UAS-NPY.GFP* was a gift from Iain Robinson (Plymouth University) (58). *UAS-lacZ*, *UAS-mito.GFP*, *D42-GAL4* and *CCAP-GAL4* were obtained from the Bloomington *Drosophila* Stock Centre (Bloomington, IN).

### Dissection and imaging of axonal transport

Analysis of axonal transport was performed live on third instar larvae as previously described (45,59). Briefly, larvae were pinned at each end dorsal side up to a Sylgard (Sigma 761028) slide and covered in dissection solution: 128 mm NaCl, 1 mm EGTA, 4 mm MgCl_2_, 2 mm KCl, 5 mm HEPES and 36 mm sucrose, adjusted to pH 7.2. Larvae were cut along the dorsal midline using micro-dissection scissors, the sides pinned back and the internal organs removed. Movies were taken using an Olympus FV1000 fluoview confocal microscope with a 60× water immersion lens (NA 0.90 Olympus LUMPFL). Images were captured at a rate of 1 frame per 5 s for 100 frames for mitochondria, or 1 frame per 0.5 s for 100 frames for vesicles. *CCAP-GAL4* was used as it expresses in a very sparse population of cells which secrete the neuropeptide CCAP (Crustacean Cardio-Active Peptide). These neurons send out a single axon in the segmental nerve thus facilitating precise imaging of transport in an individual axon.

### Analysis of axonal transport

Movies were processed and analyzed using the ImageJ software as previously described to produce kymographs (45). These were used to manually score cargo as anterograde, retrograde or stationary. Scoring was undertaken blind by two independent researchers (K.R.B. and V.K.G.) and only concordant scores were retained.

### Eclosion

Pupae were placed in groups of 25 to a vial and the proportion of adult flies that emerged was assessed. Animals from at least three replicate crosses were analyzed.

### Larval locomotion

Control and experimental crosses were established at 25 °C and transferred to fresh vials every 2 days. Vials containing wandering third instar larvae were coded by an independent researcher, placed at 23 °C and left to acclimatize for 2 h. Individual larvae were taken and rinsed in distilled water to remove any residual food, placed on a 2% agarose plate under a viewing microscope and left to acclimatize for 5 s. The number of peristaltic movements in 2 min was counted by direct observation. Animals from at least three replicate crosses were analyzed.

### Adult climbing

Male flies between 0 and 3 days post-eclosion were placed at 23 °C to acclimatize for 1 h. The flies were then transferred to the climbing tubes at a maximum number of 25 per tube for a further 1 h. Flies were introduced into a negative geotaxis counter-current apparatus and given 10 s to climb at each position for total of five attempts. A climbing index score was generated based on the number of flies at each of the positions. The score for each genotype within an experiment was normalized to the control. Animals from at least three replicate crosses were analyzed. For the longitudinal study of the effects of age-related neurodegeneration on climbing ability, male flies were aged in cohorts and the climbing assay was performed at set time points as indicated. Between assays, the flies were transferred every 2 days into fresh vials with no more than 25 flies per vial.

### Statistical analysis

Calculations were performed using GraphPad Prism 6.0. Adult climbing analysis is not normally distributed so the data were analyzed using Kruskal–Wallis non-parametric test with Dunn’s correction for multiple comparisons. All other quantifications passed the D’Agostino & Pearson omnibus normality test. For axonal transport, eclosion and larval crawling significance was determined by one-way analysis of variance (ANOVA) with the Sidak multiple comparison test (being more powerful than Bonferroni’s correction).
